# LC-MS-Based Metabolomics Study of Marine Bacterial Secondary Metabolite and Antibiotic Production in *Salinispora arenicola*

**DOI:** 10.3390/md13010249

**Published:** 2015-01-07

**Authors:** Utpal Bose, Amitha K. Hewavitharana, Yi Kai Ng, Paul Nicholas Shaw, John A. Fuerst, Mark P. Hodson

**Affiliations:** 1 School of Pharmacy, The University of Queensland, Brisbane, Queensland 4072, Australia; E-Mails: utpal.bose@uqconnect.edu.au (U.B.); a.hewavitharana@pharmacy.uq.edu.au (A.K.H.); n.shaw@pharmacy.uq.edu.au (P.N.S.); 2 School of Chemistry and Molecular Biosciences, The University of Queensland, Brisbane, Queensland 4072, Australia; E-Mails: Yi.ng@uqconnect.edu.au (Y.K.N.); j.fuerst@uq.edu.au (J.A.F.); 3 Metabolomics Australia, Australian Institute for Bioengineering and Nanotechnology, The University of Queensland, Brisbane, Queensland 4072, Australia

**Keywords:** *Salinispora arenicola*, salt concentration, antibiotic, metabolomics, secondary metabolites, liquid chromatography, mass spectrometry, multivariate analysis

## Abstract

An LC-MS-based metabolomics approach was used to characterise the variation in secondary metabolite production due to changes in the salt content of the growth media as well as across different growth periods (incubation times). We used metabolomics as a tool to investigate the production of rifamycins (antibiotics) and other secondary metabolites in the obligate marine actinobacterial species *Salinispora arenicola*, isolated from Great Barrier Reef (GBR) sponges, at two defined salt concentrations and over three different incubation periods. The results indicated that a 14 day incubation period is optimal for the maximum production of rifamycin B, whereas rifamycin S and W achieve their maximum concentration at 29 days. A “chemical profile” link between the days of incubation and the salt concentration of the growth medium was shown to exist and reliably represents a critical point for selection of growth medium and harvest time.

## 1. Introduction

The marine environment presents a unique combination of environmental factors such as salinity, pressure, low temperatures and issues of nutrition availability. When compared with their terrestrial counterparts, marine organisms require distinctive metabolic capabilities in order to adapt and thrive in such an environment. Within the past few decades, a large number of bioactive compounds have been isolated from marine organisms and several review articles have been published in this area [1,2,3].

The identification of salinosporamide A, an antitumor agent derived from obligate marine actinobacteria, has been shown to be correlated with different growth patterns on a number of salt-based media such as sodium sulphate-, sodium chloride- and potassium chloride-based formulations [4,5]. Thus, it is important to optimise the salt concentration while culturing marine bacteria to understand how marine organisms respond to different salinity conditions and produce secondary metabolites. Although production has been optimised for anticancer compounds in *Salinispora* [5], the metabolic response to similarly changed conditions during anti-bacterial rifamycin antibiotic production in this organism remains unexplored.

In addition to the discovery of new secondary metabolites from marine sources, studies have also focused on the physiological characteristics of marine microorganism and their adaptation to the marine environment, particularly Gram-negative bacteria from marine sources [6]. For example, the marine bacterium *Vibrio alginolyticus* requires sodium ions for acidification of the cytoplasm via a Na^+^/H^+^ antiport channel in order to support pH homeostasis and growth [6]. Similarly, studies on halotolerant marine fungal species have shown that marine-derived fungal metabolite production can be sensitive to seawater concentration, and this sensitivity may have implications for the drug production process [7]. A recent study of the marine-derived fungus *Spicaria elegans* showed that a high salt concentration is integral to the induction and production of new and chlorinated compounds in these halotolerant fungi [8]. Similar results were also reported for filamentous fungi where new secondary metabolites were produced, with new bioactivities, when salt concentrations were altered [9]. Although several studies have been performed on the growth requirement of different ion concentrations in Gram-negative bacteria or in fungi [7,8], similar investigations of Gram-positive bacteria are limited [9] and are particularly lacking for marine actinomycetes [10].

The concentration of sodium ions in the marine environment required for the growth of marine organisms is critical [4]. Such a requirement has been subsequently linked to the osmoregulatory mechanisms that signal the production of polyols and amino compounds in combination with increasing the concentration of cytoplasmic ions [7]. In Gram negative marine bacteria the control of osmolarity and sodium requirement has been found to be linked to electron transport and the expression of sodium pumping respiratory NADH dehydrogenase (sodium quinone reductase) Nqr [11]. More recently a comparative genomic study of *Salinispora* spp. has shown that the acquisition of marine adaptation genes (MAGs) represents a major contribution to marine adaptation while gene loss of a related MAG coding for the large conductance mechanosensitive channel (*mscL*) is proposed to play a role in the inability of *Salinispora* species to grow in deionised (DI) water-based media in laboratory culture [12]. This latter study by Penn and co-workers reveals that MAGs are associated with electron transport, sodium and ABC transporters, as well as channels and pores [12]. The ionic requirements of marine bacterial growth can also include calcium and magnesium [5], but the genetic and metabolic implications of these requirements are unknown.

Members of the genus *Salinispora* are obligate marine actinobacteria isolated from tropical and sub-tropical marine sediments, tropical sponges, algae and ascidians [13,14,15]. To date, three closely related species have been reported from this genus: *S. tropica*, *S. arenicola* and *S. pacifica* [16,17]. These bacterial species produce a large number of pharmaceutically-relevant secondary metabolites, for instance anticancer (salinosporamide A), antibacterial (rifamycins), anti-inflammatory (cyclomarin D) and antimalarial (salinosporamide A) products [18,19,20]. *Salinispora* was described as the first obligate marine actinomycetes genus based on a requirement for seawater to achieve growth in a complex medium [5,21], revealing that members of this genus fail to grow when seawater in the medium is replaced with DI water [5]. It has recently been discovered that *Salinispora* spp. are capable of growth in low sodium concentration media, such as 5 mM Na^+^, if an appropriate osmotic environment is provided [10]. Nonetheless, absolute requirements for sodium can be replaced with another two ions, either potassium or lithium, to support maximal growth [10]. Although *S. tropica* and *S. pacifica* can grow in lithium-based media, *S. arenicola* exhibits slow growth in such media [22].

Rifamycins are a group of polyketide antibiotics belonging to the family of ansamycins produced by a group of soil-derived actinomycetes of the species *Amycolatopsis mediterranei* [23]. These compounds elicit their antibacterial activity via the specific inhibition of RNA synthesis, binding to the β-subunit of RNA polymerase [24]. Rifamycin-related compounds have also been reported to be produced by *S. arenicola* isolated from Great Barrier Reef (GBR) marine sponges [15,25,26,27,28]. Kim and co-workers have suggested that horizontal gene transfer (HGT) between *Amycolatopsis* and *Salinispora* may have occurred, possibly explaining the origin of this particular class of compounds in *S. arenicola* [15]. Culture age and growth phase are known to influence production of rifamycin by *Salinispora arenicola* [26], and growth in a low-salinity medium can result in doubling of rifamycin yield [27]. Previously, several studies have reported the production of salinosporamide A in *S. tropica* with varying salt type and concentration [4,5,22].

Metabolomics is the comprehensive analysis of the biochemical content of cells, tissues or biofluids, usually from analysis of extracts [29]. Typically metabolomics experiments have utilised NMR- and/or MS-based analytical techniques to explore the metabolite content of experimental samples. Liquid Chromatography-Quadrupole Time of Flight-Mass Spectrometry (LC-QToF-MS) has received much attention in recent years for microbial metabolic fingerprinting studies, as well as in many other fields of biology [27,29,30,31]. In previous work we used HPLC-DAD to assess rifamycin production in model strain M413 across 43 days of growth, as well as a preliminary investigation of three model strains (M413, SW15 and SW17) grown in media at two NaCl salt concentrations (1% and 3%) at three time periods [27]. This HPLC-based analysis suggested a distinction in the chemotype UV spectra of secondary metabolites for *S. arenicola* strains grown in 1% NaCl relative to those grown in 3% NaCl, and additional compounds identified in 1% NaCl-grown 14-day cultures of strain M413 included rifamycin biosynthesis intermediates such as protorifamycins, rifamycin W derivatives, and rifamycin B and rifamycin S end-products of the rifamycin pathway [27]. However, the chemotype compounds found in cultures of 1% NaCl-grown strain M413 were not identified beyond the rifamycin pathway compounds in that study. In the present study, LC-MS-based metabolomics was used to identify the variation of chemical metabolite profiles of the three *S. arenicola* strains grown in media of different salinities in more detail, using chemoinformatic approaches such as PCA and OPLS-DA, to assess non-rifamycin compounds as well as to assess rifamycin pathway compounds and confirm conclusions made on the basis of HPLC-based analysis regarding rifamycins. In addition LC-MS-based analysis provides a window into compound identification by furnishing analyte-related searchable information such as accurate mass measurement and fragmentation patterns.

## 2. Results

### 2.1. Production of Rifamycins at Three Time Points of Bacterial Growth

**Figure 1:**
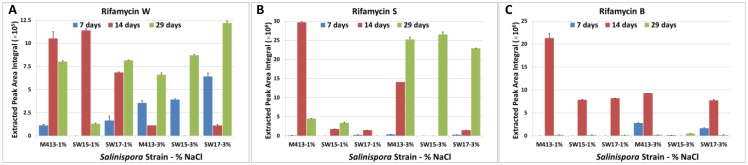
Effect of salt concentrations on production of (**A**) rifamycin W, (**B**) rifamycin S and (**C**) rifamycin B in three different time points (7, 14 and 29 days of incubation). Colours represent incubation time (days) as shown in the key. Column height indicates group mean peak area, error bars are standard deviation. NB *N* = 2 for M413-1% at 14 days; *N* = 1 for M413-3% at 14 days.

In previous work (Ng *et al.*, 2014) [27], HPLC-UV was used to monitor the production of rifamycins in broth fermentation of strain M413 over a 43 day time period. To investigate whether there were differences in the production of rifamycins at three specific incubation time points (7, 14 and 29 days), the production of three rifamycin analogues (rifamycin W, S and B) was monitored in all samples. These three compounds were previously reported from this species [28,32] and recognised as species-specific markers [14]. Identification of these three compounds was based on results of previous studies and by reference to authentic standards [18,28,32]. Results showed that the production of rifamycin W increased to a maximal level in 1% *w/v* NaCl medium after 14 days for two strains (M413 and SW15), while all three strains generated a minimal rifamycin W level at the same time point in 3% w/v of NaCl medium (Figure 1A). However, an upward trend of rifamycin W production was found during 29 days of incubation with the 3% NaCl medium. Although rifamycin W is consistently produced by M413 and SW17 in both 1% and 3% NaCl medium at the three incubation time points, this study was unable to detect any rifamycin W production in SW15 at 1% NaCl medium and at 3% NaCl medium at the 7 and 14 day time points respectively.

Time-course analysis of the production of rifamycin S showed that incubation of SW15 in 1% and 3% salt concentration medium did not produce detectable rifamycin W at the 7 day time point or when this strain was cultured in 3% salt concentration at the 14 day time point (Figure 1B). All the strains grown in 3% salt concentration showed an upward trend in rifamycin production at the 29-day time point, while strains grown in the 1% salt concentration medium showed a decrease in rifamycin S production during the same culture time period.

In this study, production of rifamycin B was not detected in 1% NaCl medium when the incubation time was 7 days (Figure 1C). Figure 1C shows that rifamycin B can be produced maximally after 14 days of incubation and then a decrease was observed at the later time points for M413 and SW17. Although all the strains showed similar patterns of rifamycin B production, strain SW15 in 3% NaCl produced a barely detectable amount of this compound.

### 2.2. Comparison of Secondary Metabolite Production in Three Strains (M413, SW15 and SW17) at Two Salt Concentrations (1% and 3% NaCl) at Three Time Points (7, 14 and 29 Days)

**Figure 2:**
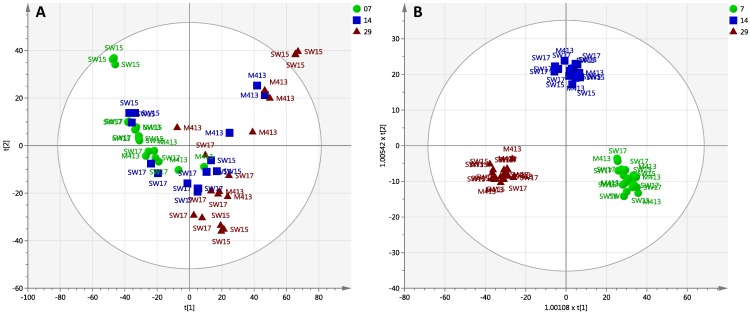
Effects of different salt concentrations and different time periods of incubation within three *S. arenicola* strains. (**A**) Principal component analysis (PCA) scores plot, PC1 (t[1]) *versus* PC2 (t[2]) showing the variation in the chemical profiles *S. arenicola* strains (green, 7 days incubation), (blue, 14 days incubation) and (red, 29 days incubation). Each symbol represents one bacterial sample described by all detected features (metabolites); (**B**) Orthogonal projection to latent structures-discriminant analysis (OPLS-DA) scores plot of predictive components t[1] *versus* t[2] showing the supervised separation between the three sample classes based upon days of incubation. The ellipses shown in A and B represents the Hotelling’s T^2^ 95% confidence interval for the multivariate data. Data are log10 transformed and mean centred. Labels indicate the *S. arenicola* strain. Scores plots of the supervision of the data by strain and salt concentration are shown in Supplementary Figure S1A,B.

The effects of salt concentration and harvesting/incubation time on the LC-MS profiles of *S. arenicola* were studied. To investigate these effects, the total dataset (all extracted compounds detected within the *m/z* range of 100–1700; 2737 detected features) was first evaluated using PCA to identify any outliers and assess any groupings or trends. The PCA scores plot shows the separation of samples based on their harvesting time points—the scores for the first two components of the model are shown in Figure 2A. A three component PCA model explains 38% of the variance in the dataset. Samples harvested after 14 days and 29 days show similarities in their chemical profiles, whereas samples harvested after 7 days incubation show a distinctly different pattern of metabolites.

Orthogonal projection to latent structures-discriminant analysis (OPLS-DA) was then used to refine the model fit and to partition the variance into predictive (ion differences related to days of incubation) and orthogonal sources (ion differences unrelated to days of incubation). The OPLS-DA model shows the complete separation between three different time points of incubation. The first two predictive components are plotted in (Figure 2B); 19% of the variance was related to days of incubation, whereas 29.1% of the variance is unrelated to days of incubation (first of four orthogonal components).

#### 2.2.1. Day 7 Time Point

**Figure 3:**
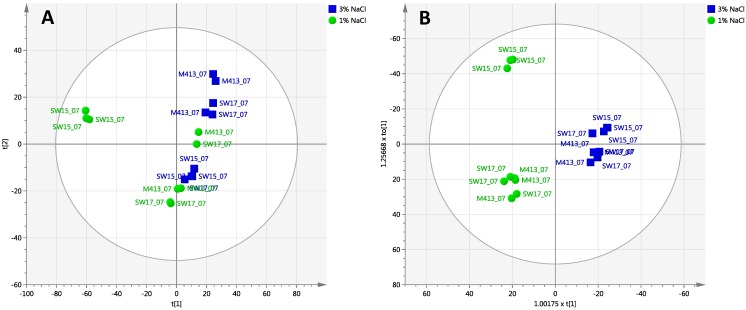

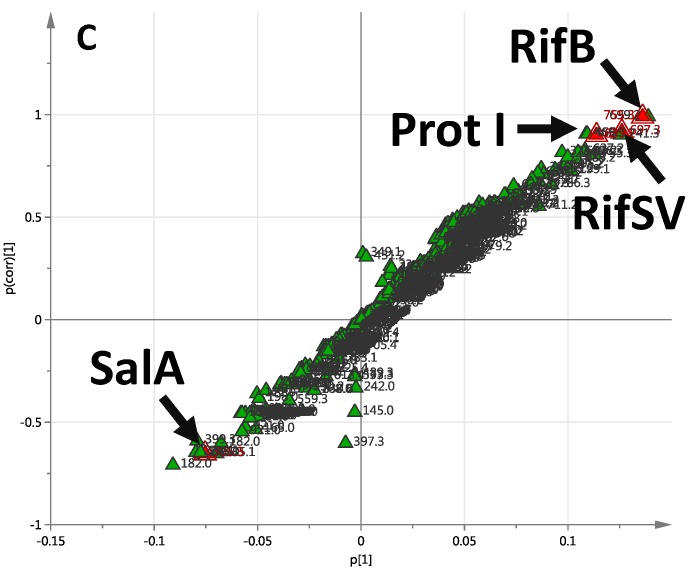
Chemoinformatic analyses of *S. arenicola* strains after 7 days growth in SYP-1% NaCl and SYP-3% NaCl. (**A**) PCA scores plot of PC1 *versus* PC2 generated from day 7 samples (*N* = 18); (**B**) OPLS-DA scores plot generated from day 7 samples (*N* = 18), supervised by % NaCl. Further analysis of individual strains are presented in Supplementary Figure S2A–C; (**C**) Loadings S-plot derived from (**B**). The S-plot shows the relationship between the correlation p(corr) and the covariance (p) of the discriminating component of the OPLS-DA model. Data are log10 transformed and mean centred. Red highlighted variables are, from left to right: SalA—Saliniketal A; Prot I—Protorifamycin I; RifSV—Rifamycin SV; RifB—Rifamycin B.

PCA was used for the 7 day time point as an initial step aimed at revealing compound targets to delineate the effect of salt concentration and strain variation at a single time point. A total of 18 samples (three strains, with three biological replicates, grown in two different salt concentrations) were evaluated by PCA, for which the first two scores vectors are plotted in (Figure 3A). Overall, the model consisted of 3 PCs that explain 52.6% of the variance within the dataset. Samples were coloured by 1% and 3% salt concentration, and labelled for the three different strains.

An OPLS-DA model for 7 days of incubation shows complete separation in PC1 allowing interpretation in this single component, with clear orthogonal clustering (Figure 3B). The predictive component explains 17.2% of the variability of the dataset, while 23.2% was explained by the first orthogonal component. In addition, the improved separation of subclasses (Figure 3B) demonstrates that the strains grown in media supplemented with 3% NaCl show similar metabolite patterns and therefore cluster together.

In order to assess the samples at 7 days of incubation, PCA and OPLS-DA models were used to identify patterns related to the same strains growing in two salt concentrations. Strain-based models are shown in Supplementary Figure S2A,C. Analysis of the loadings and VIP (variable importance in the projection) plots indicates that the variation in seven identifiable compounds is the main cause of separation between sample groups, partly summarized in Table 1, along with information from day 14 and day 29 analyses.

#### 2.2.2. Day 14 Time Point

**Figure 4:**
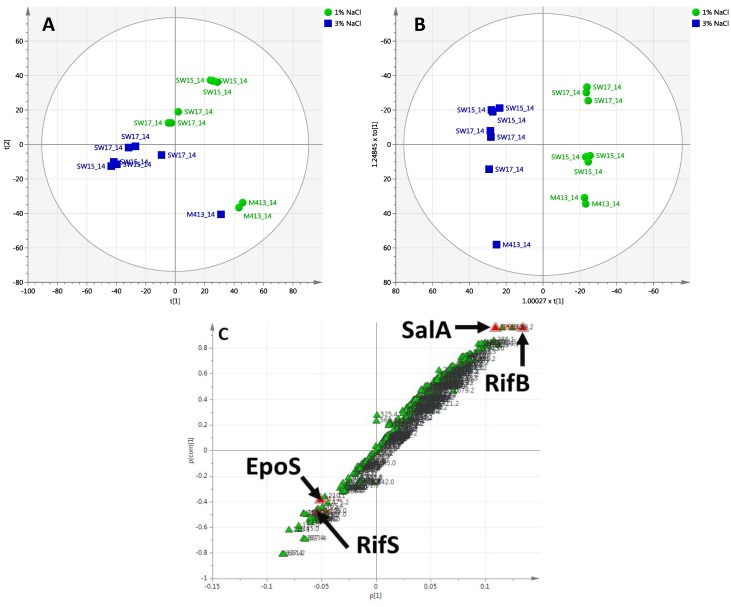
Chemoinformatic analyses of *S. arenicola* strains after 14 days growth in SYP-1% NaCl and SYP-3% NaCl. (**A**) PCA scores plot of PC1 *versus* PC2 generated from day 14 samples (*N* = 15); (**B**) OPLS-DA scores plot generated from day 14 samples (*N* = 15), supervised by % NaCl. Further analysis of individual strains are presented in Supplementary Figure S3A,B; (**C**) Loadings S-plot derived from (**B**). Data are log10 transformed and mean centred. Red highlighted variables are, from left to right: RifS—Rifamycin S; EpoS—Epoxyrifamycin S; SalA—Saliniketal A; RifB—Rifamycin B.

From the multivariate analysis of the LC-MS data at day 14, the PCA scores plot shows separation based on the salt concentration of the growth media as well as some orthogonal separation of the M413 samples (Figure 4A). OPLS-DA provided a clearer separation and was used to define features (variables) responsible for differences among the two classes related to salinity. The OPLS-DA scores plot is shown in (Figure 4B). In this model, variables that were highly relevant for explaining predicted salt effects were also identified from VIP (variable importance in the projection) and S-plot values (Supplementary Table S1). Supplementary Figure S3A,B present strain-based models, showing individual strain differences dependent upon salinity of the media. Table 1 lists the fifteen important identified variables at the 14 day incubation time after analysis of loadings and VIP plots.

#### 2.2.3. Day 29 Time Point

**Figure 5:**
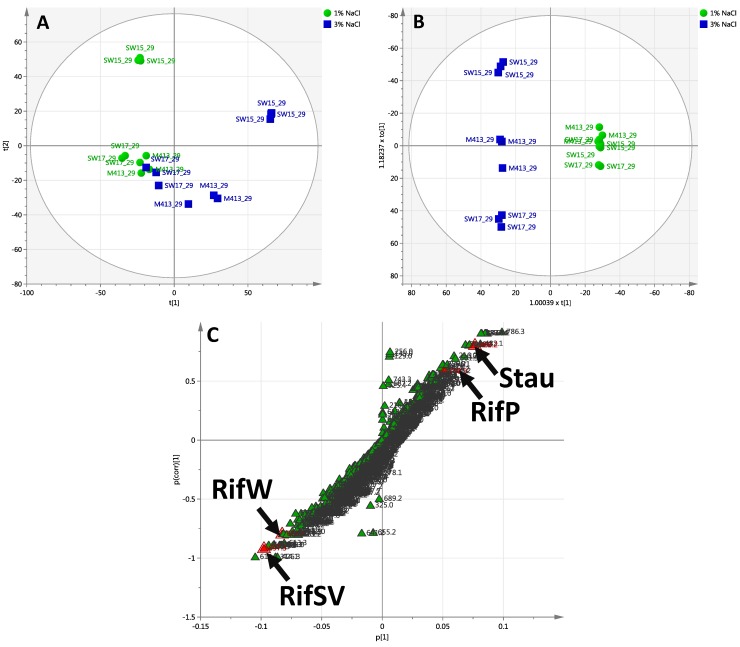
Chemoinformatic analyses of *S. arenicola* strains after 29 days growth in SYP-1% NaCl and SYP-3% NaCl. (**A**) PCA scores plot of PC1 *versus* PC2 generated from day 29 samples (*N* = 18); (**B**) OPLS-DA scores plot derived from day 29 samples (*N* = 18), supervised by % NaCl. Further analyses of individual strains are presented in Supplementary Figure S4A–C; (**C**) Loadings S-plot derived from (**B**). Data are log10 transformed and mean centred. Red highlighted variables are, from left to right: RifSV—Rifamycin SV; RifW—Rifamycin W; RifP—Rifamycin P; Stau—Staurosporine.

PCA generated a four component model that explained 62% of the variance in the dataset. The first two component scores of the model are shown in (Figure 5A). OPLS-DA was then used to refine the model fit and partition the variance into predictive and orthogonal sources. The first predictive and orthogonal components are plotted in (Figure 5B); 19% of the variance in secondary metabolites was related to two different salt concentrations (one predictive component), whereas 32.1% of the variance was unrelated to the effect of salt concentration (two orthogonal components). Model metrics are presented in Supplementary Table S1. For the comparison of two salt concentrations (1% and 3% NaCl), twelve compounds were tentatively identified and classified according to the PCDL (Table 1).

To further investigate the chemical profiles of individual strains after 29 days of incubation, PCA models were generated to delineate the differences between individual strains when grown in two different salt concentrations, presented in Supplementary Figure S4A–C. Interestingly, cycloaspeptide A, a fungus-derived cyclic peptide, was identified from the 29 days incubation period from *S. arenicola* along with other rifamycin analogues. Identification of this compound has previously been documented [33].

**Table 1:** Metabolite table summarising details of compound and matching criteria/metrics.

Molecular Formula	Score *	Mass **	Polarity	RT (min)	Mass Error (MFG, ppm)	Compound ***	Day(s) Observed
**C_38_H_49_NO_12_S**	95.35	743.2975	Negative	15.28	3.97	Awamycin	14
**C_39_H_49_NO_14_**	94.34	755.3153	Negative	8.80	0.52	Rifamycin B	7, 14
**C_38_H_46_N_2_O_11_S**	91.91	738.2822	Negative	10.16	3.50	Rifamycin P	14, 29
**C_37_H_45_NO_13_**	90.34	711.2891	Positive	10.34	2.60	18,19-Epoxyrifamycin S	14, 29
**C_22_H_37_NO_6_**	89.72	411.2621	Positive	9.40	1.44	Saliniketal B	7, 14, 29
**C_39_H_47_NO_15_**	87.34	769.2946	Negative	13.54	1.52	Rifamycin Y	14
**C_37_H_47_NO_12_**	90.27	697.3098	Negative	22.10	1.81	Rifamycin SV	7, 14, 29
**C_36_H_45_NO_12_**	68.75	683.2942	Negative	15.42	0.54	16,17 Dehydroxyrifamycin G	29
**C_37_H_45_NO_12_**	84.34	695.2942	Negative	22.61	3.43	Rifamycin S	7, 14, 29
**C_22_H_37_NO_5_**	81.72	395.2672	Positive	12.58	1.44	Saliniketal A	7, 14, 29
**C_12_H_12_N_2_O_2_**	86.05	216.0899	Negative	5.86	1.36	Mansouramycin A	7, 14
**C_28_H_26_N_4_O_3_**	74.68	466.2005	Positive	18.47	3.19	Staurosporine	7, 14, 29
**C_35_H_45_NO_11_**	95.67	655.2993	Negative	7.25	1.1	Rifamycin W	14, 29
**C_35_H_45_NO_10_**	95.58	639.3043	Negative	9.19	1.0	Protorifamycin I	14, 29
**C_36_H_43_N_5_O_6_**	94.23	641.3213	Positive	13.72	1.94	Cycloaspeptide A	14, 29
**C_35_H_43_NO_11_**	67.65	653.2836	Negative	8.62	1.84	(34aR)-Rifamycin W hemiacetal	14, 29

^*^ Overall score calculated from database search as described in the method. ^**^ Neutral accurate mass calculated for each compound. ^***^ Compound identification performed through the in-house accurate mass database match, MS/MS fragmentation and reference standard (rifamycin B, S, SV and cycloaspeptide A). RT = retention time.

## 3. Discussion

In the marine environment, salt concentration plays a central role in the growth of microorganisms [34] and their secondary metabolite production [7]. In this study, metabolomics was used as a tool to begin to understand how the pattern of secondary metabolite (and therefore antibiotic) production in the obligate marine *S. arenicola* species might be affected by salt concentration. The approach of integrating LC-MS-based profiling with bacterial secondary metabolite production should have broad applicability to study bacterial species with respect to the effects of environmental factors such as salt concentration on the synthesis of metabolites such as antibiotics.

In this study, the production of three rifamycin compounds was monitored over the time course of incubation (29 days) and at two different salt concentrations. In the initial stages of this study using LC-MS, the production of rifamycin derivatives was detected in all the strains, as found for some rifamycin compounds assayed in a previous study from our laboratory using only HPLC and diode array detection [27]. The production of rifamycin W varied according to the NaCl concentration and days of incubation. In previous studies, it was reported that rifamycin W is a precursor for rifamycin S, SV, B, L and Y [23,35]. Another study reported that rifamycin B production is higher in all the strains when the strains were grown for 4 weeks and gradually converted to SV and S over time [32]. Bannerjee and co-workers also reported that rifamycin B is a precursor for rifamycin Y and other rifamycin analogues, and the enzyme rifamycin oxidase facilitates the biotransformation process from rifamycin B to rifamycin S to provide stability and antibacterial activity of this compound [36]. The aforementioned studies and the results therein support the present study, where rifamycin B production is decreased at 29 days, while the production of rifamycin W and S increased. It is possible that the interconversion of rifamycin B to S and W by rifamycin oxidase may be the cause for the increased concentration of these compounds. Further studies of this enzyme are necessary to reveal the detailed mechanism of interconversion.

Chemoinformatic analyses using PCA and OPLS-DA enabled correlation and delineation of chemical profiles with salinity conditions of growth over time. These data indicate that rifamycin analogues and saliniketals are among the most important chemical discriminators contributing to differences between the three strains as well as amongst growth conditions. Insights gleaned from such analyses can now be leveraged in studies of the design and optimisation of future antibiotic production in *Salinispora* species, thereby allowing deeper investigation of the selection of optimal salt media for production (perhaps even a range of salt media may be developed for optimising different compounds).

In addition to rifamycins we found other compounds to be responsible for experimental differences between the sample sets. For example staurosporine, which has previously been found in *Streptomyces* [14], *S. arenicola* [14] and *S. pacifica* [35]. Studies relating to *staD* genes associated with staurosporine biosynthesis in two *Salinispora* species reveal two distinct lineages possibly inherited from a common ancestor [37]. Although the close relationship of the *staD* gene sequence in *Streptomyces* spp. offers strong evidence that this pathway has been horizontally exchanged, it is not clear at which point this occurred [13,37]. Another compound, cycloaspeptide A, was also determined to be important in differences observed at days 14 and 29. Previous studies found that sponge-derived *Pseudomonas* spp. produce cycloaspeptides [38]. One explanation for the occurrence of this class of compound from *Salinispora* spp. may be horizontal gene transfer (HGT) between two adjacent species [33]. It is known that sponges act as a reservoir for microbial species and there exists ongoing competition between adjacent species. Thus, the possibility of gene exchange may occur and subsequently confer an adaptive benefit, allowing survival in a particular environment. We found consistent production of saliniketals along with rifamycins in different salt concentrations throughout the study period. This is consistent with the known shared synthetic pathway for saliniketals and rifamycins [35]. Previous findings indicate that saliniketals A and B are produced exclusively to *S. arenicola* species and may also represent species-specific markers along with rifamycins to distinguish *S. arenicola* from two other *Salinispora* species [28].

The observation from LC-MS-based metabolomics of distinct chemical profiles of *Salinispora* strains grown in 1% and 3% NaCl broth is consistent with a previous study of rifamycin production in *Salinispora* at two different salt concentrations based only on HPLC-UV-DAD-analysis [27]. However, the LC-MS study reported herein identifies further compounds with which the differences can be correlated. Relative to the production of rifamycin-like compounds, both the strains and salt concentrations have effects on the production of secondary metabolites and indicate a greater degree of diversity. Our results may have implications for the range of habitats with respect to salinity which this *Salinispora* strain can inhabit competitively. Low salinity may effectively constitute a stressful environment condition for an obligate marine *Salinispora* bacterium, and a response to such stress may include alterations in the metabolome and secondary metabolite spectrum. These differences in chemical profile based on salt concentration might conceivably have an impact on the ability of such bacteria to compete within a sponge microbial community subject to salinity stress [12]. Low salinity events associated with freshwater flooding are known to adversely affect and even kill Great Barrier Reef invertebrates [39], so reef sponges and their microbial communities may well be periodically subjected to low salinity stress.

## 4. Experimental Section

### 4.1. Sample Preparation

Three *S. arenicola* strains: M413, SW15 and SW 17 were grown in Starch-Yeast-Peptone media supplemented with either 1% NaCl (SYP-1% NaCl) or 3% NaCl (SYP-3% NaCl) in three replicate shake flask cultures using methods described by Ng *et al* [27]. Each of the *Salinispora* strains were inoculated into Erlenmeyer flasks containing 50 mL of SYP-1% NaCl and SYP-3% NaCl broth respectively. The flasks were incubated on an orbital shaker (Bioline, Smeaton Grange, New South Wales, Australia) at 28 °C for 29 days. From the complete sample set of 378 samples (3 strains; 3 replicates; 14 time points; 3 salt concentrations) a subset of 54 samples (3 strains; 3 replicates; 3 time points; 2 salt concentrations) was selected for LC-QToF-MS analysis. At 7, 14 and 29 days after inoculation, 5 mL of the broth culture was removed from the flask. As a negative control, one flask of sterile non-inoculated broth was incubated simultaneously and 5 mL of this broth was extracted after 29 days of incubation. Five millilitres of ethyl acetate was then added and the mixture vortex mixed for 1 min followed by centrifugation at 1000 × *g* for 3 min at room temperature to separate the ethyl acetate and the aqueous broth layers. Four millilitres of the upper ethyl acetate layer was then transferred to a clean polypropylene centrifuge tube. The ethyl acetate was evaporated using a vacuum centrifuge (Savant Instruments, Hicksville, NY, USA) for 1 h at a low drying rate. The dried extract was reconstituted in 600 μL of 20% v/v methanol/water and frozen at −80 °C until analysis.

### 4.2. UHPLC-QToF-MS Analysis

The samples were analysed using an Agilent UHPLC-Q-ToF-MS system (Agilent Technologies, Santa Clara, CA, USA) comprising a 1290 UHPLC coupled to a 6520 Accurate-Mass Quadrupole Time-of-Flight Mass Spectrometer (QToF-MS, Agilent Technologies, Santa Clara, CA, USA) in fast polarity switching mode from *m*/*z* 100 to 1700 for all samples at a scan rate of 0.8 cycles/s. Instrument resolution was 9000–11,700 across the data acquisition range. This mass range enabled the inclusion of two reference compounds, a lock mass solution including purine (C_5_H_4_N_4_ at *m*/*z* 121.050873, 10 µmol·L^−1^) and hexakis (1*H*, 1*H*, 3*H*-tetrafluropentoxy)-phosphazene (C_18_H_18_O_6_N_3_P_3_F_24_ at *m/z* 922.009798, 2 µmol·L^−1^). Chromatographic separation was achieved using a Phenomenex Gemini-NX C18 HPLC column (150 mm × 2.0 mm, 3 µm, Phenomenex, Lane Cove, NSW, Australia). The mobile phase consisted of (A) water with 5 mM ammonium acetate (UniVar Analytical reagents, Sydney, Australia) and (B) acetonitrile (ACN) (LabScan Analytical Science, Taren Point, Australia). In all HPLC runs the elution gradient started at 80% A: 20% B increasing to 0% A: 100% B over 40 minutes, followed by a 5 minute hold and 20 minutes re-equilibration period.

A sample volume of 20 μL was injected for each HPLC run. The HPLC run contained blanks, a sample-relevant standard solution [40], and pooled samples [41] intercalated throughout the HPLC run to control for any acquisition-dependent variation. The samples and standards were filtered using a 0.2 µm PTFE membrane filter (Phenomenex, Torrance, CA, USA) before analysis.

Multimode (Electrospray Ionisation (ESI) (Agilent Technologies, Santa Clara, CA, USA) and Atmospheric Pressure Chemical Ionization (APCI) with Fast Polarity Switching (FPS) (Agilent Technologies, Santa Clara, CA, USA) was used to ionise and detect compounds after chromatographic separation. The general parameters of the MS1 mixed mode source were as follows: capillary voltage 3500 V, nebulizer pressure 30 psi, drying gas 7.0 L·min^−1^, gas temperature 300 °C, vaporizer 200 V, voltage charge 2000 V; negative-ion mode capillary voltage 2500 V, corona negative 15.0 V, fragmentor 175 V, skimmer1 65.0 V, octopole RF Peak 750 V; positive ion mode capillary voltage 2500 V, corona positive 4.0 V, fragmentor 175 V, skimmer1 65.0 V and octopole RF Peak 750 V. Data processing was performed using Agilent MassHunter Qualitative software (Version B.05.00, Agilent Technologies, Santa Clara, CA, USA).

### 4.3. Data Processing and Molecular Formula Generation

The Molecular Feature Extractor (MFE) algorithm within MassHunter Qualitative analysis software (Agilent Technologies, Santa Clara, CA, USA) was used to extract chemically qualified molecular features from the LC-QToF-MS data files. For empirical formula generation, the Molecular Formula Generator (MFG) algorithm was used. This algorithm uses a wide range of MS information, for instance accurate mass measurements, adduct formation, multimer formation and isotope patterns to generate a list of candidate compounds. The maximum elemental composition C_60_H_120_O_30_N_30_S_5_Cl_3_Br_3_ was used to generate formulae. MFG can automatically eliminate unlikely candidate compounds and rank the putative molecular formulae according to their mass deviation, isotopic pattern accuracy and elemental composition. *S. arenicola* extracts from three different time points (7, 14, 29 days), two ASW concentrations (1% and 3% NaCl) and three different strains (M413, SW15 and SW17) were evaluated separately by multivariate statistical analysis. Feature-extracted sample files were transferred into Agilent GeneSpring software version 12.0 (Agilent Technologies, Santa Clara, CA, USA) for alignment and to compile the data matrix.

### 4.4. Chemometric Analyses and Compound Identification

*S. arenicola* extracts from three different time points (7, 14, 29 days), two ASW concentrations (1% and 3% NaCl) and three different strains (M413, SW15 and SW17) were evaluated separately by multivariate statistical analysis. Feature-extracted sample files were transferred into Agilent GeneSpring software version 12.0 (Agilent Technologies, Santa Clara, CA, USA) for alignment and to compile the data matrix. The data matrix (2737 variables; 51 observations—three observations from day 14 were omitted from the analysis due to unsuccessful extraction/acquisition) was imported into SIMCA-P+ version 13.0 (MKS Umetrics AB, Umeå, Sweden) for multivariate data analysis, using primarily principal component analysis (PCA) and orthogonal projection to latent structures-discriminant analysis (OPLS-DA). For more information relating to these methods see Trygg *et al.* (2007) and Bylesjö *et al.* (2006) [42,43]. Data were pre-treated by log10 transformation and mean centring prior to analysis. Data were also analysed using pareto and unit variance scaling but scaling was deemed superfluous for feature selection in this dataset and thus only mean centred analysis/results are shown. For OPLS-DA models a minimum threshold of VIP>1 was used for variable selection (= “importance”) [44].

Metabolite peaks were assigned by searching their accurate mass, referencing an in-house PCDL (personal compound database library). This PCDL was compiled in-house to screen secondary metabolites from *Salinispora* (and other) species used for future studies on marine and terrestrial chemical ecology. The PCDL includes information relating mainly to secondary metabolites from marine sources, primarily retrieved from research articles and review papers [45,46,47,48,49,50,51,52,53,54,55,56,57,58,59,60,61,62,63,64]. Inclusion to the PCDL was then followed up by compound curation and annotation using public database sources such as SciFinder (http://www.cas.org/products/scifinder), PubChem (http://pubchem.ncbi.nlm.nih.gov/) and ChemSpider (http://www.chemspider.com/). These resources were used to retrieve molecular formula and other chemical information—the PCDL platform calculates the monoisotopic mass for each entity and has links to PubChem and ChemSpider to facilitate further information retrieval. To date approximately 7200 compounds have been included in the PCDL.

Compound identification in this study was carried out either by comparison to previously acquired MS and MS/MS data or by interrogating the PCDL using the *m*/*z* values of the mined compounds from metabolic profiles via molecular feature extraction (MFE) and molecular formula generation (MFG). The search parameters implemented were as follows: mass tolerance (accurate mass) ≤ 5 ppm, maximum number of peaks to search when peaks are not specified graphically = 5, charge carriers (positive ions) = H^+^, K^+^, Na^+^, negative ions = H loss, Cl^−^, Br^−^ and HCOO^−^ and neutral loss = −H_2_O. The scoring algorithm for database searches uses not only accurate mass, but also isotope abundance and spacing. The mass position of the M + 1 and M + 2 isotopes is calculated based on the number and types of elements contributing to them, and the mass spacing from the M to the M + 1 and M + 2 isotopes can be measured with low- to sub-ppm accuracy and provide confidence for compound identification.

## 5. Conclusions

The study described here is empowered by the application of metabolomic profiling, using liquid chromatography coupled to high-resolution mass spectrometry, enabling the optimisation of salt media concentration with regards to rifamycin production in obligate marine bacteria in *S. arenicola*. The obtained metabolomics analysis and “chemical profile” link between the days of incubation and different salt concentration of the growth medium is sufficiently direct and reliable to represent a robust critical point for media selection and harvesting time. Thus, this study allowed the elucidation of the impact of some of the multiple factors that are important for secondary metabolite production in marine-derived actinobacterial species capable of bioactive compound synthesis. The findings presented here establish a foundation for the future development of optimum growth media for the production of bioactive compounds in *S. arenicola* (and other marine bacterial species), and for the design of media to be used in screening programs for the identification of new compounds from marine bacteria.

## Supplementary Files

Supplementary File 1
