# Supplementary material for: LC-MS-Based Metabolomics Study of Marine Bacterial Secondary Metabolite and Antibiotic Production in Salinispora arenicola
Source: Mar Drugs. 2015 Jan 7;13(1):249–66. doi: 10.3390/md13010249 (PMC4306935; doi:10.3390/md13010249)
Supplement: Supplementary File 1 [file marinedrugs-13-00249-s001.pdf]

Supplementary Information

Table S1. Summary of information and metrics for the multivariate models used for feature selection.

Model Comparison	Number of Variables	Model Type	R^2X	Q^2	VIP > 1
All samples (7, 14 and 29 days)	1390	PCA	0.56	0.34	
All samples (7, 14 and 29 days)	1390	OPLS-DA	0.97	0.87	389
7 days (1% *vs*. 3% NaCl)	502	PCA	0.44	0.28	
7 days (1% *vs*. 3% NaCl)	504	OPLS-DA	0.98	0.76	163
7 days M413 (1% *vs*. 3%)	229	PCA	0.47	0.26	
7 days M413 (1% *vs*. 3%)	231	OPLS-DA	0.89	0.99	100
7 days M415 (1% *vs*. 3%)	323	PCA	0.72	0.59	
7 days M415 (1% *vs*. 3%)	325	OPLS-DA	0.72	0.99	173
7 days M417 (1% *vs*. 3%)	240	PCA	0.44	0.21	
7 days M417 (1% *vs*. 3%)	242	OPLS-DA	0.86	0.88	107
14 days (1% Vs 3% NaCl)	479	PCA	0.70	0.46	
14 days (1% Vs 3% NaCl)	481	OPLS-DA	0.64	0.58	173
14 days M415 (1% *vs*. 3%)	305	PCA	0.85	0.79	
14 days M415 (1% *vs*. 3%)	307	OPLS-DA	0.85	0.99	199
14 days M417 (1% *vs*. 3%)	254	PCA	0.52	0.33	
14 days M417 (1% *vs*. 3%)	256	OPLS-DA	0.82	0.96	123
29 days (1% *vs*. 3% NaCl)	849	PCA	0.54	0.34	
29 days (1% *vs*. 3% NaCl)	851	OPLS-DA	0.51	0.99	234
29 days M413 (1% *vs*. 3%)	469	PCA	0.44	0.30	
29 days M413 (1% *vs*. 3%)	471	OPLS-DA	0.81	0.92	207
29 days M415 (1% *vs*. 3%)	625	PCA	0.78	0.68	
29 days M415 (1% *vs*. 3%)	627	OPLS-DA	0.78	0.99	327
29 days M417 (1% *vs*. 3%)	361	PCA	0.46	0.19	
29 days M417 (1% *vs*. 3%)	363	OPLS-DA	0.80	0.98	150

R^2X—"goodness of fit" parameter, varies between 0 and 1, and provides a quantitative measure of the explained variation, where 1 = all variance explained; Q^2—"goodness of prediction" parameter, varies between 0 and 1, and provides a cross-validated measure of the predictive power of the model, where 1 = complete prediction; VIP—"variable importance in the projection" parameter—variables with VIP>1 are considered the most relevant for explaining the differences denoted by Y, which for OPLS-DA is the grouping variable outlined by the model comparison e.g., 1% NaCl Vs 3% NaCl [44].

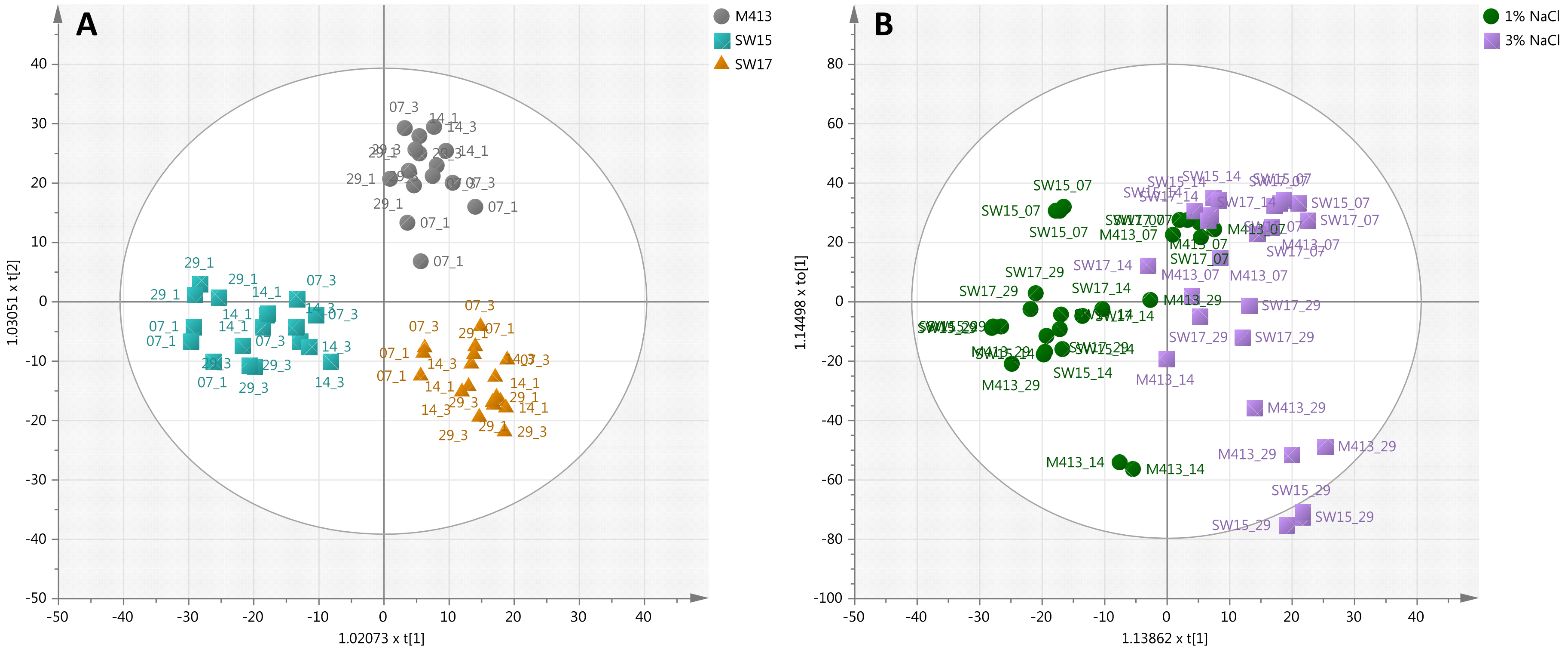

Figure S1. OPLS-DA models of the complete data set (*N* = 51), indicating the supervised "separation" based upon (**A**) *S. arenicola* strain and (**B**) % NaCl.

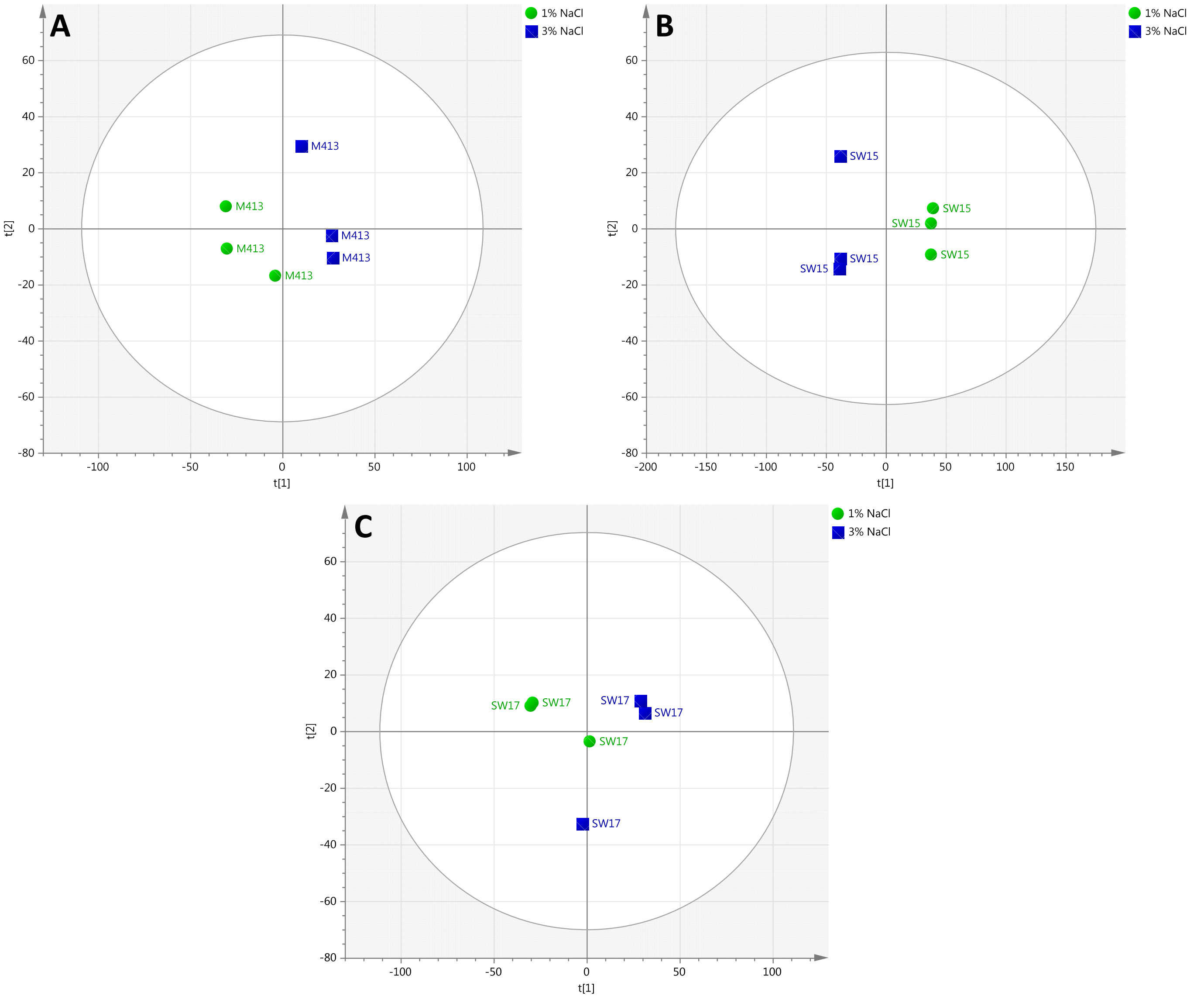

Figure S2. PCA models of day 7 samples (*N* = 18) indicating strain-based differences for (**A**) M413; (**B**) SW15 and (**C**) SW17.

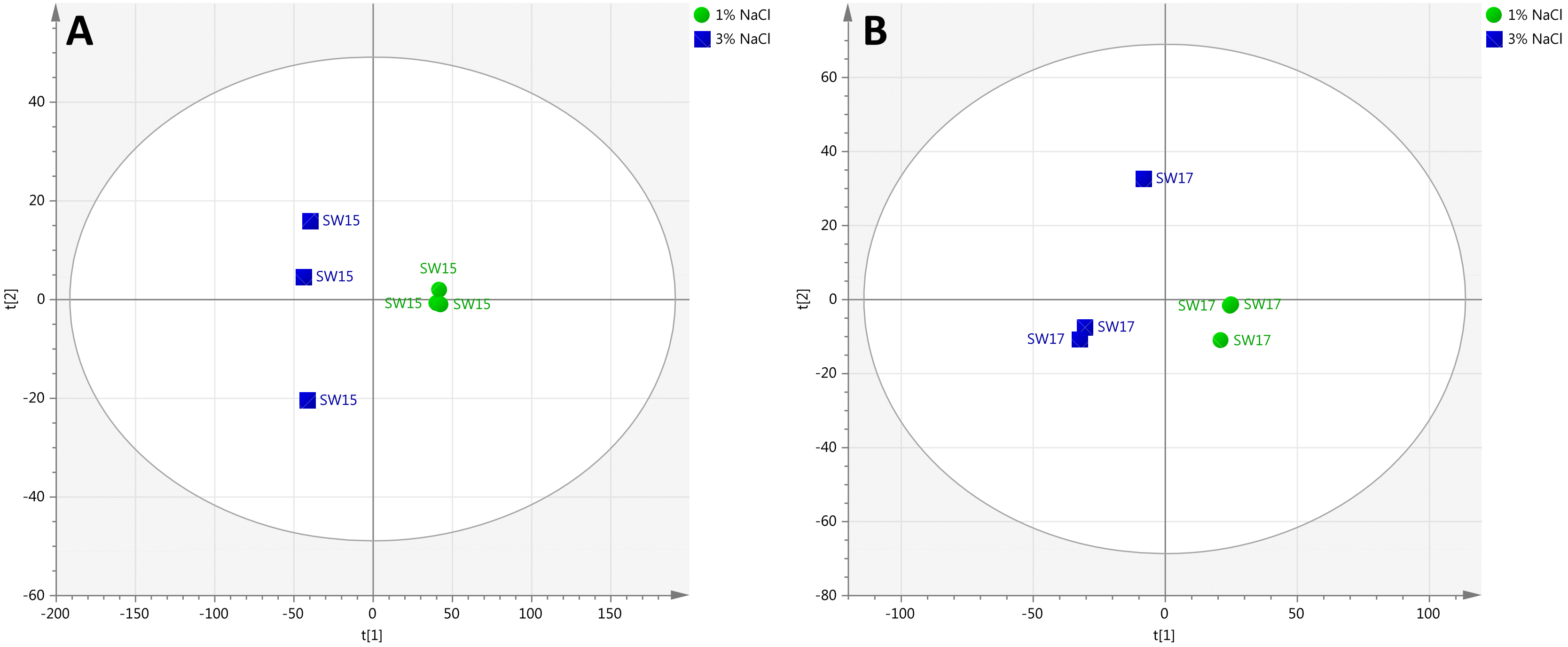

Figure S3. PCA models of day 14 samples (N = 15) indicating strain-based differences for (**A**) SW15 and (**B**) SW17.

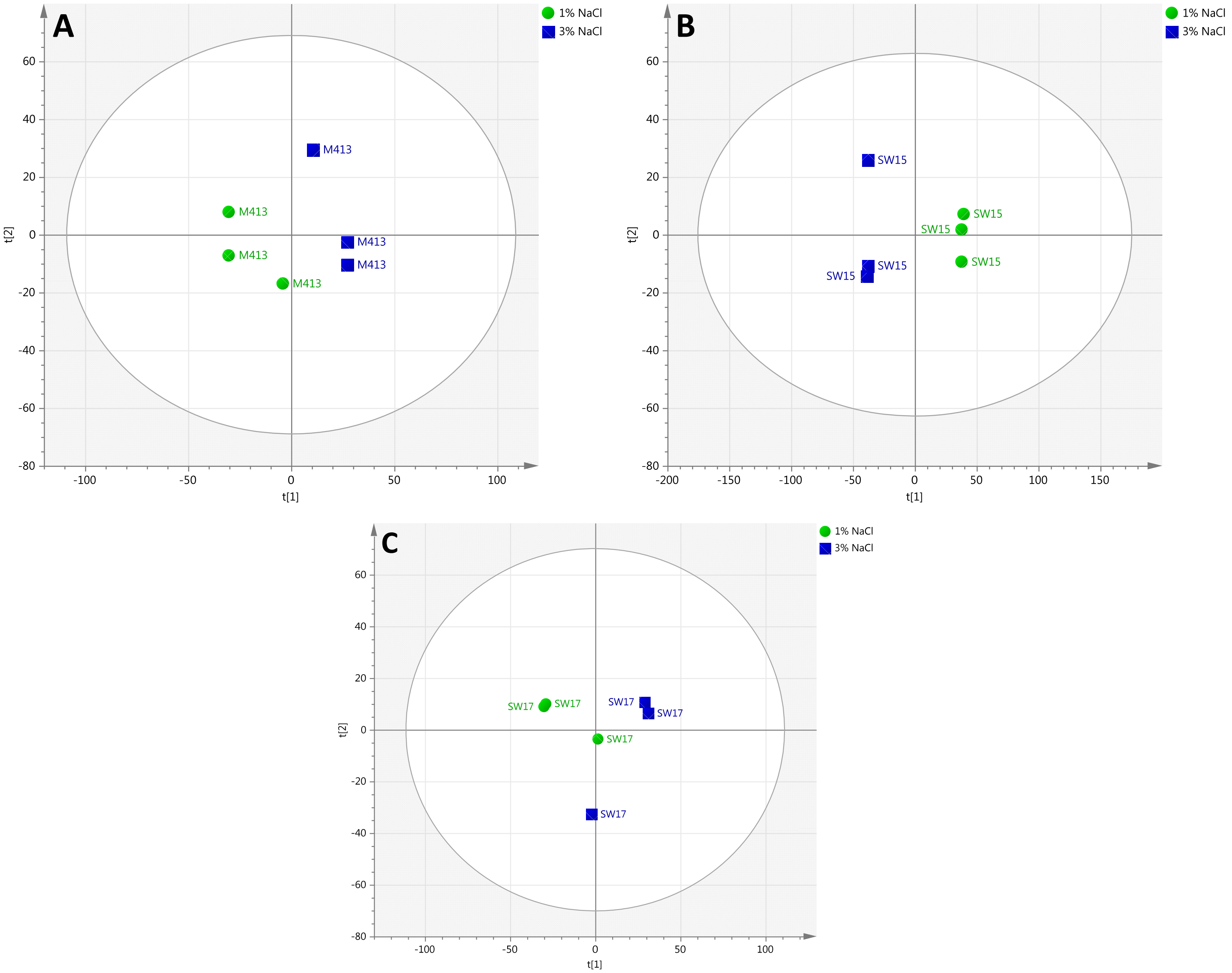

Figure S4. PCA models of day 29 samples (*N* = 18) indicating strain-based differences for (**A**) M413; (**B**) SW15 and (**C**) SW17.

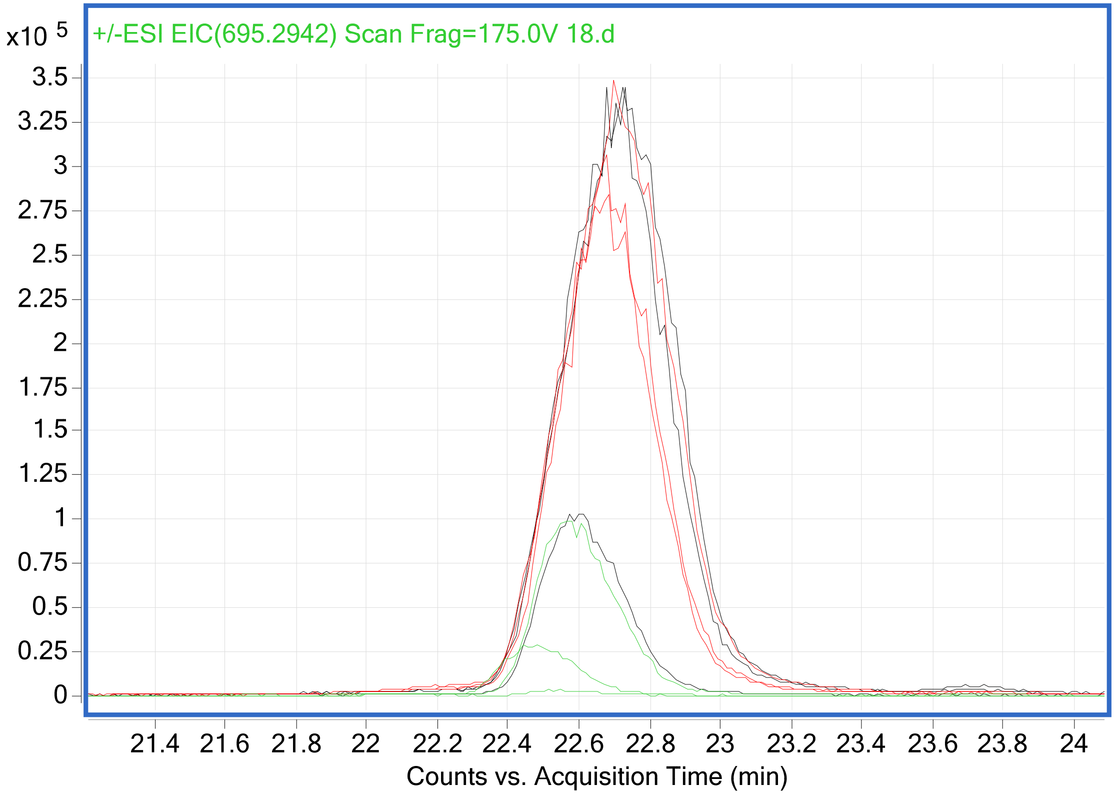

Figure S5. Extracted ion chromatograms showing strain-related differences for Rifamycin S day 29; 3% NaCl; M413; SW15; SW17.

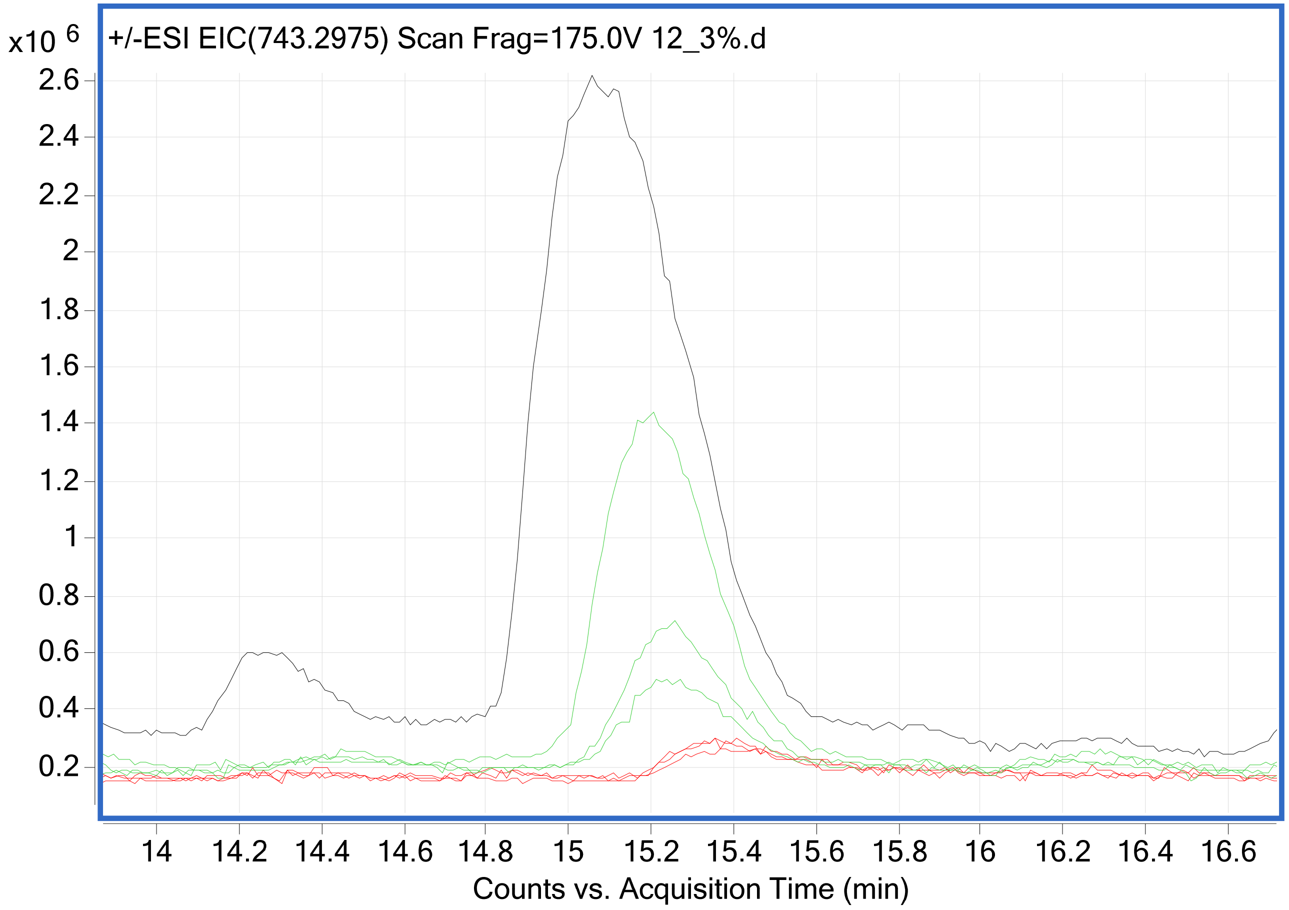

Figure S6. Extracted ion chromatograms showing strain-related differences for Awamycin day 14; 3% NaCl; M413; SW15; SW17.

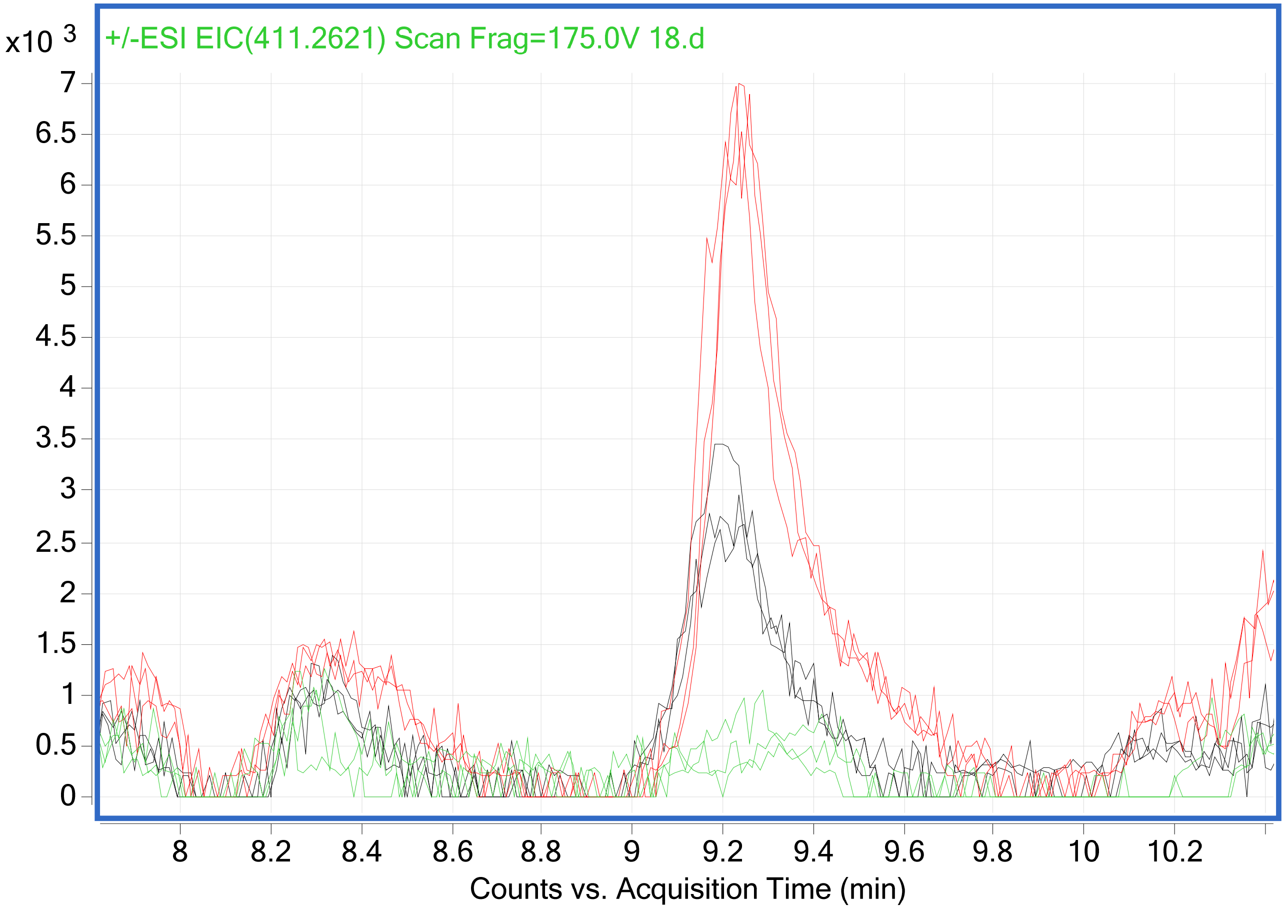

Figure S7. Extracted ion chromatograms showing strain-related differences for Saliniketal B day 29; 3% NaCl; M413; SW15; SW17.

© 2015 by the authors; licensee MDPI, Basel, Switzerland. This article is an open access article distributed under the terms and conditions of the Creative Commons Attribution license (http://creativecommons.org/licenses/by/4.0/).
